# Thirty years of contact angles reveal universal design rules for wetting control

**DOI:** 10.1038/s41598-026-40965-x

**Published:** 2026-02-23

**Authors:** Amir Karimdoost Yasuri

**Affiliations:** Department of Mechanical Engineering, Lorestan University, Khorramabad, Iran

**Keywords:** Wettability, Contact angle, Superhydrophobicity, Surface design, Data curation, Materials informatics, Meta-analysis, Chemistry, Engineering, Materials science

## Abstract

Wettability, commonly quantified by the static contact angle (θ), governs critical interfacial phenomena including anti-icing, self-cleaning, adhesion control, and lubrication. Although conceptual thresholds for superhydrophilic and superhydrophobic states are widely cited, their empirical validation across material classes has remained limited by the absence of a comprehensive, rigorously verified dataset spanning diverse surfaces and liquids. Here, we compile and systematically analyze 110 curated static contact-angle measurements reported between 1995 and 2025, encompassing polymers, metals, oxides, self-assembled monolayers (SAMs), and micro-/nano-textured surfaces measured with multiple probe liquids. Our meta-analysis quantitatively confirms the existence of universal critical thresholds, with θ ≲ 20° defining superhydrophilicity and θ ≳ 150° defining superhydrophobicity. Crucially, for textured and hierarchical surfaces, these limits emerge as geometry-dominated properties, persisting across material classes and largely independent of intrinsic surface chemistry. These validated thresholds establish clear, principle-driven design rules for engineering functional wetting behavior, moving beyond trial-and-error approaches. The resulting dataset provides a reliable benchmark for the community, supporting predictive wettability design, cross-study meta-analysis, and the development of data-driven and machine-learning models without the need for repetitive experimental measurements.

## Introduction

Wettability, quantified by the static contact angle (θ) of a liquid droplet on a solid surface, is a fundamental interfacial property governing a wide range of technological and natural phenomena, including anti-icing, self-cleaning, adhesion control, lubrication, and biointerfacing^1^. The ability to reliably predict and engineer target wetting states is therefore critical across disciplines spanning materials science, surface engineering, and biomedical applications.

Central to wettability design are the extreme regimes of superhydrophilicity and superhydrophobicity, commonly associated with contact-angle thresholds of θ < 20° and θ > 150°, respectively. However, these states require more precise definition than static contact-angle values alone. Superhydrophilicity is rigorously characterized by the *rapid* spreading of a liquid droplet to an equilibrium contact angle approaching 0–10° within sub-second timescales, rather than slow capillary imbibition or absorption observed in porous or fibrous media^2^. Similarly, superhydrophobicity is not defined solely by a high static contact angle, but must also be accompanied by low contact-angle hysteresis and small roll-off or sliding angles, indicative of a stable Cassie–Baxter wetting state^3^. Apparent high contact angles arising from partially impregnated or metastable “rose-petal” Cassie states must therefore be distinguished from true Lotus-effect behavior^4^.

Classical wetting theory, encapsulated by the Wenzel and Cassie–Baxter models, predicts that surface texture amplifies intrinsic chemical wettability, suggesting that extreme wetting states emerge from a combination of surface chemistry and geometry^5^. Despite this conceptual framework, a substantial gap remains between theory and practical, cross-material design rules. Reported contact-angle values in the literature are highly fragmented, typically restricted to individual material–liquid pairs measured under non-standardized conditions. Variability in surface preparation, droplet volume, ambient environment, and measurement protocols further complicates direct comparison across studies^6^. As a result, a fundamental question has remained unresolved: do universal, quantitative thresholds for superwetting behavior exist across material classes, and to what extent does geometry dominate over chemistry?

It is also important to emphasize that static contact angle alone does not fully characterize functional wetting performance. True superhydrophobic behavior requires low contact-angle hysteresis and low sliding angles, which govern droplet mobility, self-cleaning efficiency, and resistance to fouling. However, such dynamic metrics are inconsistently reported in the historical literature, limiting their utility for large-scale comparative analysis. Consequently, the present meta-analysis focuses on static equilibrium contact angles as a unifying and widely available descriptor, while recognizing that extreme static θ values represent a necessary—but not sufficient—condition for practical superwetting functionality.

Extreme wetting states ultimately arise from the synergistic coupling of surface chemistry and hierarchical micro/nano-texture. Chemistry establishes the intrinsic Young’s contact angle of a flat surface, while texture amplifies this baseline through geometric effects, enabling access to superhydrophilic or superhydrophobic regimes that are otherwise unattainable on smooth substrates.

To address the lack of consolidated evidence, we present a curated and verified 30-year dataset comprising 110 static contact-angle measurements, spanning polymers, metals, oxides, carbon-based materials, self-assembled monolayers, and textured or nanostructured surfaces, with documented measurement conditions. The primary contribution of this work is not the introduction of new experimental data, but the systematic meta-analysis of this verified ensemble to extract universal physical principles. Our analysis robustly confirms the existence of sharp critical thresholds—approximately 20° and 150°—that define the transition to superhydrophilic and superhydrophobic behavior, respectively. Crucially, for textured surfaces these thresholds are largely independent of the underlying surface chemistry, demonstrating that geometry plays the dominant role in enabling extreme wetting states.

These findings provide a concise, geometry-driven design rulebook for engineering functional wettability across material classes. Beyond immediate design guidance, the curated dataset establishes a validated benchmark for predictive modeling, machine-learning-assisted surface discovery, and future meta-analyses, enabling progress in wettability engineering without reliance on repetitive experimental measurements.

## Methods

### Data collection

We compiled a curated and verified dataset of 110 static contact-angle (θ) measurements reported between 1995 and 2025 from peer-reviewed literature. The dataset spans a broad range of solid surfaces, including polymers, metals, metal oxides, carbon-based materials, self-assembled monolayers (SAMs), and textured or nanostructured substrates. Measurements encompass multiple probe liquids—primarily water, but also glycerol, ethylene glycol, diiodomethane, and selected oils—enabling cross-liquid comparisons of wettability trends.

For surface–liquid systems in which a classical three-phase contact line is ill-defined—such as superhydrophilic surfaces (θ ≲ 10°), liquid-infused surfaces, and interfaces governed by Neumann-triangle wetting—the reported values correspond to *apparent* contact angles rather than true equilibrium Young angles. These entries are explicitly identified and treated separately in the analysis to avoid conflating fundamentally distinct wetting mechanisms with rigid solid–liquid contact-angle behavior.

To ensure reliability, comparability, and reproducibility, only literature reports satisfying all of the following criteria were included:

1. Clearly reported static contact angles, measured using established and standardized protocols (e.g., sessile-drop or equivalent methods), with explicit distinction between advancing, receding, and equilibrium values where available^7^.
2. Documented surface preparation and characterization, including cleaning procedures, chemical functionalization, coating deposition, or micro-/nano-texturing methods, enabling reproducible interpretation of surface chemistry and geometry.
3. Controlled and reported measurement conditions, including droplet volume, ambient temperature, humidity, and contact-angle goniometry instrumentation or analysis methodology.

Entries lacking sufficient methodological detail, relying on secondary citation without access to primary measurements, or reporting ambiguous or non-reproducible experimental conditions were excluded. This stringent curation minimizes systematic bias arising from inconsistent measurement protocols and ensures that the dataset represents a high-confidence reference suitable for cross-material and cross-liquid comparison.

The resulting dataset provides a robust empirical foundation for identifying universal wettability thresholds, disentangling the respective roles of surface chemistry and texture, and formulating transferable, geometry-driven design rules for functional surfaces^1,5^.

The curation goal was not statistical exhaustiveness, but physical representativeness across major material classes and wetting regimes. The dataset is designed to cover the conceptual design space (superhydrophilic, hydrophobic, superhydrophobic, lubricant-infused) with verified entries, enabling identification of fundamental geometry-driven trends.

### Data organization

All verified entries were systematically organized into a standardized, machine-readable master table to ensure clarity, comparability, and reproducible analysis. Each row corresponds to a unique solid–liquid pair and reports the following information:

(1) solid surface identity and class, including polymers, metals, oxides, carbon-based materials, self-assembled monolayers (SAMs), and textured or hierarchically structured surfaces; (2) probe liquid, including water, glycerol, ethylene glycol, diiodomethane, and selected oils; (3) static contact angle θ (degrees), reported as equilibrium values where specified, or as apparent values for non-classical wetting systems; (4) measurement conditions and contextual notes, including droplet volume, ambient conditions, surface preparation or modification, and measurement methodology; and (5) primary literature reference. Entries are sorted primarily by increasing contact angle and secondarily by publication year, facilitating visualization of wetting-state transitions across the full wettability spectrum. This structured organization enables direct cross-comparison between material classes and liquid types, supports identification of critical thresholds for extreme wetting regimes, and allows optional secondary analyses linking θ to surface chemistry, roughness, or hierarchical architecture.

By maintaining a consistent and transparent data structure, Table 1 serves not only as the analytical foundation for this study but also as a validated reference dataset suitable for future modeling efforts, machine-learning-assisted wettability prediction, and rational surface design without requiring repeated experimental measurements^1,5^.

### Data analysis

The curated dataset was analyzed to identify universal trends and critical thresholds governing wettability across materials and surface classes. Static contact angles were first grouped by material category (polymers, metals, oxides, carbon-based materials, SAMs, and textured or nanostructured surfaces) and by liquid type, enabling assessment of both chemistry- and liquid-dependent effects on θ^1^.

A threshold-based analysis was then applied, classifying surfaces with θ < 20° as *superhydrophilic* and those with θ > 150° as *superhydrophobic*. These thresholds are consistent with classical wetting theory and widely accepted experimental criteria, and are further supported here by the emergence of a bimodal distribution in θ across the full dataset^5,8^. The sharp clustering of values near these limits indicates that extreme wetting states represent distinct physical regimes rather than a continuous extrapolation of moderate wettability.

Importantly, comparative analysis across flat and textured surfaces reveals that surface geometry plays a dominant role in accessing these extreme regimes. While intrinsic surface chemistry sets the baseline Young’s contact angle, hierarchical micro-/nano-texture strongly amplifies this intrinsic wettability, enabling chemically moderate surfaces to achieve θ > 150° or θ ≈ 0° when appropriately structured. This observation holds across polymers, metals, oxides, and carbon-based materials, indicating that the identified thresholds are largely chemistry-independent for textured surfaces.

Where sufficient auxiliary information was available, optional correlations between θ and surface parameters—such as roughness scale, hierarchical architecture, SAM coverage, and chemical functionalization—were examined qualitatively. These analyses confirm that hierarchical roughness is a necessary condition for robust superhydrophobicity, while chemistry primarily governs stability, durability, and aging effects^9^.

Overall, this analytical framework provides a cross-material, physics-based understanding of wettability, enabling extraction of transferable design principles for functional surfaces in anti-icing, self-cleaning, drag reduction, and adhesion-controlled applications, without reliance on trial-and-error experimentation.

## Results

### Dataset overview

Table 1 compiles a verified dataset of 110 static contact-angle (θ) measurements reported between 1995 and 2025, encompassing a broad range of solid surfaces and probe liquids. The dataset spans polymers, metals, oxides, carbon-based materials, self-assembled monolayers (SAMs), cellulose-based substrates, and micro-/nano-textured or hierarchically structured surfaces, providing comprehensive coverage of both flat and engineered interfaces.

Measurements were performed using multiple liquids—including water, glycerol, ethylene glycol, diiodomethane, and selected oils—allowing assessment of wettability across polar and nonpolar systems. For classical solid–liquid–vapor configurations, reported values correspond to equilibrium or quasi-equilibrium static contact angles. For systems in which a well-defined three-phase contact line does not strictly exist—such as superhydrophilic surfaces (θ ≲ 10°), lubricant-infused surfaces, and oil–water–solid interfaces—the reported values represent apparent contact angles and are interpreted accordingly.

The collected θ values span the entire wettability spectrum, from complete wetting (θ ≈ 0°) to extreme non-wetting states exceeding 170°. Notably, despite the diversity of materials, liquids, and surface treatments, the dataset exhibits pronounced clustering near two limits—θ < 20° and θ ≥ 150°—foreshadowing the emergence of universal thresholds for superhydrophilic and superhydrophobic behavior. This breadth and internal consistency make the dataset a robust foundation for identifying cross-material trends and for developing geometry-driven design rules for functional wettability.

**Table 1:** Verified static contact angles of selected solid–liquid pairs (1995–2025).

Solid surface	Liquid	θ (°) Static	Conditions/notes	Refs.
TiO_2_ (polycrystalline film)	Water	0	After UV illumination (λ < 380 nm, 1.1 mW cm⁻²).	^10^
TiO_2_ (with certain % SiO_2_)	Water	0	Film prepared with SiO_2_ acquires this property after UV illumination.	^10^
Titania-coated glass / Plain glass	Water	5	After treatment with UV/ozone, immersion in aqua regia, or heating above 500 °C.	^11^
TiO₂ (anatase, UV treated)	Water	5	Superhydrophilic after UV irradiation	^12^
Iα (010), Iα (11̅0), Iβ (010), Iβ (110), Human-made cellulose II & III_I faces	Water	11	Values determined via molecular dynamics simulation.	^13^
PET film (plasma treated)	Water	23.5 ± 1.7	After plasma treatment at 20 kHz, CDA 0.6%, 7 repetitions	^14^
Iβ (11̅0) crystal face	Water	32	Value determined via molecular dynamics simulation.	^13^
Iβ (100) crystal face	Water	34	Value determined via molecular dynamics simulation.	^13^
TiO_2_ nanotube surfaces	Water	39.1	Increased hydrophilicity compared to non-anodized Ti (75.9°).	^15^
Titanium (various treatments)	Distilled water	10–73	Contact angle depends on surface treatment. Hydrophobic surfaces correlate with rutile-type oxide.	^16^
PMMA	Diiodomethane	43.2	Equilibrium/Young	^17^
Iα (001) crystal face	Water	48	Near the hydrophilic/hydrophobic boundary.	^13^
Anodized Ti (TiO₂ NTs)	Water	49	Hydrophilic surface after anodic oxidation	^18^
PET (incipient alkaline hydrolysis)	Water	50	Predicted limiting advancing contact angle under specific NaOH treatment	^19^
PMMA	Formamide	56.7	Equilibrium/Young	^17^
PMMA	Ethylene Glycol	57	Equilibrium/Young	^17^
Iron (Fe)	Distilled water	57	Freshly polished surface. Measured via sessile drop method.	^20^
Glass surfaces	Water	20–95	Wettability altered by treatment with dichlorooctamethyltetrasiloxane (Surfasil).	^21^
Aluminum (polished)	Water	58	Smooth metal, ambient	^20^
Molybdenum (Mo)	Distilled water	58	Used for validation of derived equation.	^20^
PMMA	Glycerol	58 ± 2	Sessile drop, surface energy probe liquid	^22^
PET film	Water	59 ± 1.1	Before atmospheric-pressure plasma treatment	^14^
Graphite (freshly exfoliated)	Water	60 ± 13	Fresh exfoliation; experimental reference matched by modeling	^23^
Stainless steel (SS316L)	Ethylene glycol	60 ± 2	sessile drop, Value is stable across a temperature range of 25–80 °C	^24^
Titanium (Ti)	Distilled water	63	Freshly polished surface. Measured via sessile drop method.	^20^
SS 316 L (biopolymer composite coating)	water	51–75	Coated samples only; FESEM, SPM, AFM, FTIR used.	^25^
Silver (Ag, polished substrate)	Water	64 ± 5	Measured distilled water on freshly polished substrates	^20^
CeO_2_ (110)	Water	64 ± 3	Orientation-dependent	^26^
Talc	Water	64.4	Equilibrium/Young	^27^
PMMA	Glycerol	66.8	Equilibrium/Young	^17^
Niobium (Nb)	Distilled water	67	Freshly polished surface. Measured via sessile drop method.	^20^
PMMA	Water	67.3 ± 1.1	100% acetic acid treatment + UV irradiation (234 nm, 30 s)	^28^
Nickel (Ni)	Distilled water	68	Freshly polished surface. Measured via sessile drop method.	^20^
Tungsten (W)	Distilled water	68	Freshly polished surface. Measured via sessile drop method.	^20^
Mercury (Hg)	Distilled water	69	Liquid metal substrate, used for validation.	^20^
PET film (untreated)	Water	70	Initial contact angle before Ar⁺ irradiation	^29^
Tin (Sn)	Distilled water	71	Freshly polished surface. Measured via sessile drop method.	^20^
PMMA	Water	72	Sessile drop 2 µL, 22 °C, surface energy probe	^7^
Silicon (Si)	Distilled water	72	Semi-metal, used for validation of derived equation.	^20^
PMMA	Water	72.8	Equilibrium/Young	^17^
Copper (oxidized)	Water	73	Freshly polished surface. Measured via sessile drop method.	^20^
Gold (Au)	Distilled water	73	Freshly polished surface. Measured via sessile drop method.	^20^
SS 316 L (SLM & cast)	NR (likely water)	59.3–86.9	Selective Laser Melted samples more hydrophilic than casted ones	^30^
Micropatterned cellulose nanocrystal (CNC) films	Water	41–106	Wettability modulated by controlling physical micropattern without changing surface chemistry.	^31^
AISI 316 L stainless steel	Water	0–150	Wettability controlled via femtosecond laser surface modification.	^32^
Non-anodized Titanium	Water	75.9	Baseline contact angle for comparison with anodized TiO_2_ nanotubes.	^15^
Graphene oxide (defective, 0–10%)	Water	70–82	Contact angle increases with defect concentration; MD simulations	^33^
Untreated Ti	Water	77	Bare titanium substrate	^18^
Graphene	Water	80	Optimized force fields consistent with experimental means	^23^
Polycarbonate (PC)	Water	82	Untreated PC substrate; sessile drop method	^34^
Graphene (fully suspended)	Water	86	Computational modeling; consistent with experimental value 85 ± 5°	^35^
Graphene (epitaxial on SiC)	Water	92	No thickness dependence; surface-energy engineering study	^36^
CeO_2_ (100)	Water	93 ± 3	Single crystal	^26^
Graphene (partial wetting transparency limit)	Water	96	Predicted highest water contact angle; theory + experiment	^37^
Graphene bilayer (misoriented, 40°)	Water	97.97 ± 1.15	Maximum angle at 40° mismatch; MD simulations	^38^
Various Substrates	Glycerol/Water Mixtures	38–160	Resonant frequency & width studied. Contact angle range provided.	^39^
PDMS (oxidized)	Water	75–125	Oxidation decreases macroscopic contact angle; MD simulations	^40^
Si wafer (ODS or FAS SAM)	Water	100	Organosilane SAMs formed at vapour/solid interface (ODS, FAS)	^41^
PET–SiOx film (FDTS SAM)	Water	101	1 H,1 H,2 H,2 H-perfluorodecyltrichlorosilane; optimal concentration 26.5 mM dm⁻²	^42^
Polypropylene (PP)	Water	104	RT, sessile drop 3 µL	^1^
SLIMS3	Latex (40% w/w)	105.7 ± 1.1	Infused with Fomblin Y-HVAC 140/13.	^43^
SLIMS2	Latex (40% w/w)	107.6 ± 0.8	Infused with Fomblin Y-LVAC 16/6	^43^
PDMS (Sylgard 184)	Water	108 ± 2	Drop ~ 2 µL, 22 °C	^44^
Dentin surface (OTS silanized)	Water	109	OTS increases CA from 51° to 109°	^45^
Si-based MEMS surface (FOTS SAM)	Water	110	Chemical vapor deposition of FOTS monolayer for adhesion control	^46^
Typical hydrophobic flat surface	Water	100–120	General reference value provided in the context of superhydrophobic coatings.	^47^
SLA Titanium (sandblasted, acid-etched, aged 4 weeks)	Water	110	Stored in dark ambient conditions. Hydrocarbon-contaminated surface.	^12^
SLA Titanium + TiO_2_-B@anatase NWs (no UV)	Water	110	Treated with nanowire suspension in the dark.	^12^
TiO_2_ nanorod thin films	Water	110 (initial) → 20 (after UV)	Reversible wettability change under UV laser irradiation.	^48^
CeO₂ (111)	Water	112 ± 3	Single-crystal surface	^26^
Pristine (untreated) PTFE	Water	113.8 ± 1.4	Conventional PTFE surface	^49^
SLIMS1	Latex (40% w/w)	115.3 ± 0.7	Infused with Krytox 101	^43^
SLIPS on TiO_2_ Nanotubes (Krytox GPL 105 Oil)	Water	118	The measured water contact angle (WCA) indicates a slippery surface.	^18^
SLIPS Ti (Final)	Water	118	Anodized, modified (5 vol% TMPSi), infused with Krytox GPL 105 Oil	^18^
H-terminated Si(111) (fluoro-hydro alkyne SAM)	Water	119	Fluoro-hydro alkyne–derived SAM; static CA reported	^50^
Polycarbonate (PC) - Laser textured	Water	120	Surface textured via CO₂ laser ablation.	^51^
Pressed Drop on Spikes	Water	130 ± 5	Transition to Wenzel state upon pressing.	^5^
Glass (OTS-treated, oil–solid interface)	Water	130	Water droplets at oil/OTS-glass interface	^52^
Lubricant-Infused Multiscale-Textured Surface	Water	130.1 ± 0.8	Chemically functionalized.	^43^
Organosilicon film (fluorination-etched)	Water	128–138	Conformity deposition + fluorination etching	^53^
SLIMS2	Water	134.1 ± 1.0	Infused with Fomblin Y-LVAC 16/6	^43^
Flat Si wafer (fluoro-polymer film)	Water	138	Fluoro-based block copolymer film; smooth surface	^54^
SrTiO_3_ nanorod arrays (oleic acid treated)	Water	140	Surface treated with oleic acid. Wettability is reversible with UV illumination.	^55^
Generic SLIPS / LIS	Water	140	The high apparent contact angle at the drop-lubricant interface facilitates easy sliding.	^56^
Wood surface (OTS modified)	Water	140.8	OTS at 2 vol % on Bi_2_O_3_-doped Si–Ti composite film	^57^
TiO_2_ nanorods on Si pyramids	Water	141	Hydrophobicity enhanced by ion irradiation-induced oxygen vacancy migration.	^58^
SS 316 L (laser-irradiated in water)	Water	142.5	Hydrophobic surface created; unpolished SS316L reference 82 ± 4°	^59^
Rough CNT-structured Si (fluoro-polymer film)	Water	150	Hierarchical roughness on CNTs enhances hydrophobicity	^54^
Biomimetic superhydrophobic surfaces (general)	Water	150	Artificial surfaces created by combining hierarchical roughness	^8^
Titanium oxide (various structures)	Water	150	High static contact angle achieved via a two-step process. Adhesion is tunable.	^60^
Cellulose paper (fluorine-free modified)	Water	150	Filter paper modified with organosilanes.	^61^
Hairy PDMS-coated glass	Water	150.5 ± 0.4	AAO-templated hierarchical PDMS	^62^
PMMA with POTS-functionalized silica nanoparticles	Water	150.0 ± 0.44	Superhydrophobic, self-cleaning plastic surface created via sol-gel process.	^63^
PMMA substrate (POTS coating)	Water	150.0 ± 0.44	Sol-gel processed surface modified with perfluorooctyltriethoxysilane	^63^
Fluorinated silica nanoparticle film	Water	151 ± 4	Superhydrophobic thin film from fluorinated SiO_2_ nanoparticles	^64^
PTFE - Plasma Etching (PE) treated	Water	152.3 ± 1.7	Treated with O_2_/Ar plasma (PE method).	^49^
SHS (Superhydrophobic Surface)	Latex (40% w/w)	153.8 ± 0.2	Base fluoroalkyl-functionalized silica/PVDF-HFP composite.	^43^
CNTs on Si micropillar arrays	Water	153–155	Hierarchical structure (micro-/nanoscale).	^65^
SLIMS1	Water	155.4 ± 0.6	Infused with Krytox 101	^43^
Micropillar array (lubricant-infused)	Water	158 ± 5	Lubricant: Decanol, surface underfilled by 1–2 μm.	^56^
Micropillar array (lubricant-infused)	Water	160 ± 3	Apparent contact angle at solid-TPCL.	^56^
Superhydrophobic coating on glass	Water	160	Optically transparent coating based on functionalized SiO_2_ nanoparticles.	^47^
SHS (Superhydrophobic Surface)	Water	161.1 ± 0.6	Base fluoroalkyl-functionalized silica/PVDF-HFP composite.	^43^
Micropillar array (lubricant-infused)	Water	166 ± 3	Decanol, surface overfilled by 32 ± 1 μm.	^56^
Modified Anodized Ti (5 vol% TMPSi)	Water	166	Superhydrophobic surface after silane modification	^18^
PDMS hierarchical surface	Water	170	Dual-scale roughness induces superhydrophobicity	^66^
PTFE - Reactive Ion Etching (RIE) treated	Water	172.5 ± 1.2	Treated with O₂/Ar plasma (RIE method)	^49^
Inverse opal (infused with FC70)	Water	174 ± 3	Surface overfilled by ~ 10–25 μm.	^56^
Inverse opal (infused with FC70)	Peanut oil	175 ± 3	Surface overfilled by ~ 10–25 μm.	^56^
Inverse opal (infused with FC70)	Hexadecane	175 ± 2	Surface overfilled by ~ 10–25 μm.	^56^

Entries are sorted primarily by increasing contact angle and secondarily by publication year.

### A curated dataset for universal wettability analysis

Our compiled dataset, presented in summary form in Table 2, now comprises 110 verified static contact angle (θ) measurements reported between 1995 and 2025. While not exhaustive, the dataset is deliberately curated to span all major material classes, surface architectures, and wetting regimes relevant to extreme wettability design.

The dominant probe liquid is water, supplemented by diiodomethane, glycerol, ethylene glycol, latex suspensions, and selected oils, enabling comparison across polar and nonpolar solid–liquid interactions. Each surface was systematically classified into one of four categories—Flat, Textured (micro-/nano-rough), Porous, or SLIPS/LIS (Slippery or Lubricant-Infused Porous Surfaces)—based on its interfacial architecture rather than chemistry alone. This classification forms the structural basis for the universal wettability analysis developed in subsequent sections.

**Table 2:** Summary of verified static water contact angles grouped by material class and surface architecture.

Material class	Surface type	Contact angle θ [°] (Range)	Representative surfaces
Polymers	Flat	67–114	PMMA, PET, PC, PDMS, pristine PTFE
	Textured	120–172	Plasma-etched PTFE, hierarchical PDMS, laser-textured PC
	Porous/composite	130–150	PMMA with functionalized silica nanoparticles, sol–gel polymer coatings
	SLIPS / LIS	105–155	SLIMS1–3, lubricant-infused polymer textures
Metals	Flat	35–82	Al, Fe, Ti, Ni, Cu, SS316L
	Textured	130–166	Laser-irradiated SS316L, anodized and silane-modified Ti
	SLIPS/LIS	118–140	SLIPS on anodized Ti, lubricant-infused metal oxides
Oxides	Flat	0–112	UV-treated TiO₂, CeO₂ single crystals, SiO₂-based glass
	Textured	110–150	TiO₂ nanorods, SrTiO₃ nanorod arrays
	Porous/infused	140–174	Inverse opal oxides (lubricant-infused)
Carbon-based	Flat	60–98	Graphite, graphene, graphene oxide
	Textured/hierarchical	150–155	CNT arrays, CNT–Si hierarchical structures
SAMs/thin coatings	Flat	100–120	OTS, FOTS, FDTS on Si or glass
	Textured/composite	128–166	Fluorinated nanoparticle films, SAM-functionalized rough oxides
Cellulose/bio-based	Flat	11–75	Cellulose crystal faces, untreated paper
	Textured/modified	106–150	Micropatterned CNC films, silane-modified paper

Reported ranges are derived exclusively from the curated dataset in Table 1 and highlight the intrinsic limits of flat surfaces and the universal role of texture and lubricant infusion in enabling extreme wetting states.

### Distribution of wettability and universal design thresholds

**Fig. 1:**
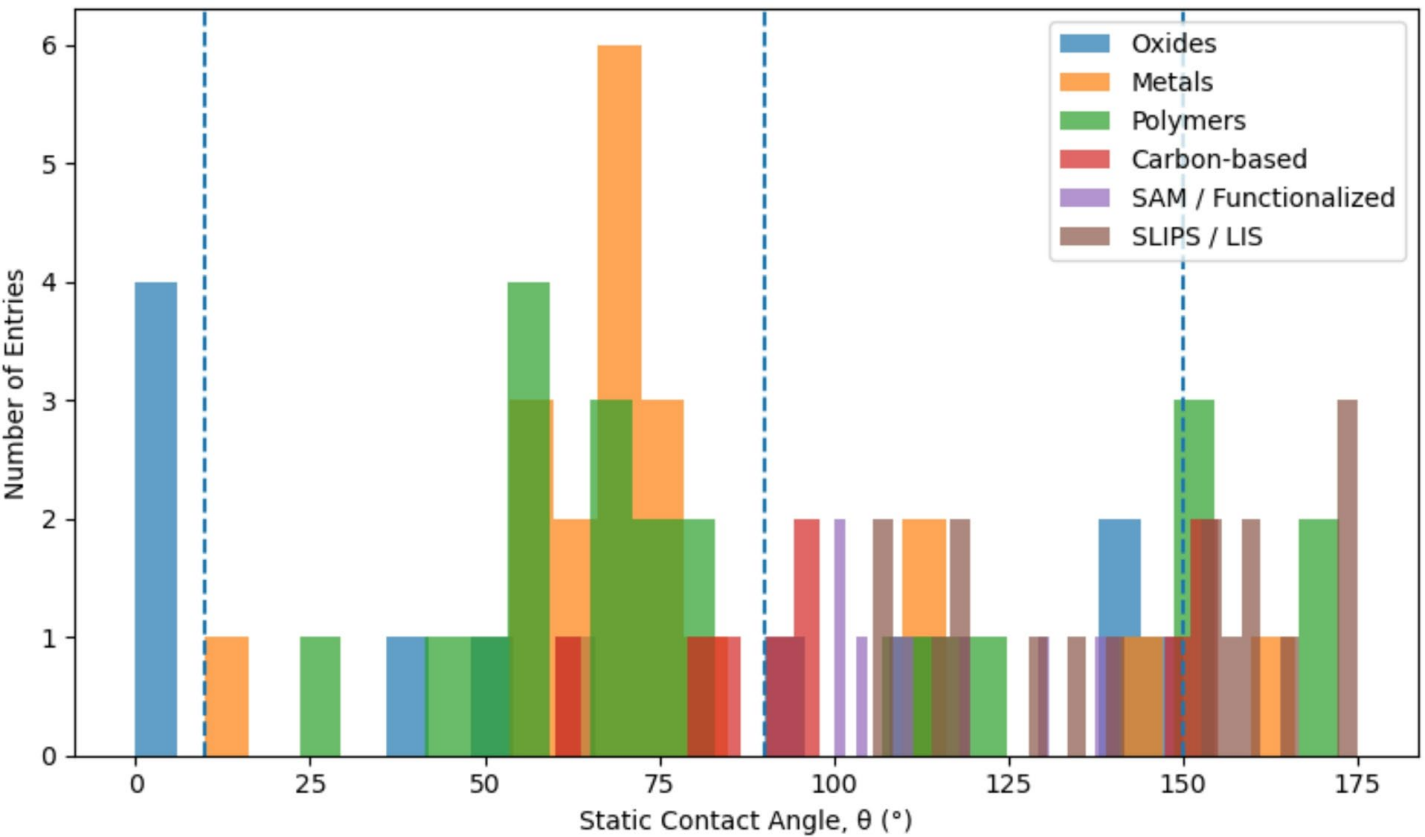
Distribution of 110 verified static contact angle measurements reported between 1995 and 2025, grouped by material class. Superhydrophilic behavior (θ ≤ 10°) corresponds to rapid equilibrium spreading on high-energy surfaces. The intermediate regime (10°–120°) is chemistry-dominated and includes polymers, metals, oxides, and SAM-functionalized surfaces. Superhydrophobic states (θ ≥ 150°) arise exclusively from surface texturing or liquid infusion, highlighting a geometry-driven transition beyond the intrinsic limits of flat-surface chemistry. Apparent contact angles measured on liquid-infused surfaces are shown separately to emphasize their distinct wetting mechanism.

The distribution of verified static contact angles compiled in this study is best described as tri-modal, corresponding to hydrophilic, hydrophobic, and superhydrophobic wetting regimes (Fig. 1). When grouped by material class, the data reveal clear and reproducible clustering behavior that is consistent across polymers, metals, oxides, and carbon-based surfaces.

The superhydrophilic regime is confined to very low contact angles (θ ≤ 10°) and corresponds to rapid equilibrium spreading, rather than slow capillary imbibition. Entries in this regime are dominated by high-energy oxide surfaces (e.g., UV-activated TiO₂, UV-ozone-treated silica) and selected porous substrates. Surfaces exhibiting gradual wicking behavior without rapid spreading are not classified as superhydrophilic. The intermediate regime (approximately 10° < θ < 120°) encompasses the majority of flat, chemically modified surfaces, including polymers, metals, oxides, and self-assembled monolayers (SAMs). This range reflects chemistry-dominated wettability, where incremental changes in surface energy lead to gradual shifts in contact angle. Importantly, the refined dataset now contains multiple verified entries in the 120°–140° range, demonstrating that this region is experimentally accessible and represents a transitional wettability space, rather than a forbidden or unstable design zone. The superhydrophobic regime (θ ≥ 150°) forms a distinct upper cluster and comprises approximately one-quarter of the dataset. Crucially, no flat, chemically homogeneous surface appears in this regime. Every surface exhibiting θ ≥ 150° relies on surface texturing, hierarchical roughness, or liquid infusion. This sharp separation confirms that exceeding the ~ 120° limit of flat-surface chemistry requires a geometry-driven transition, rather than further chemical optimization.

Vertical reference lines at θ = 10° and θ = 150° therefore delineate universal design thresholds for extreme wetting states. While surface chemistry governs wettability within the moderate regime, access to superhydrophobicity represents a fundamental shift in wetting mechanism, enabled by micro-/nano-structuring or lubricant mediation rather than incremental tuning of surface energy alone.

### The transition from chemistry-dominated to geometry-dominated wetting

**Fig. 2:**
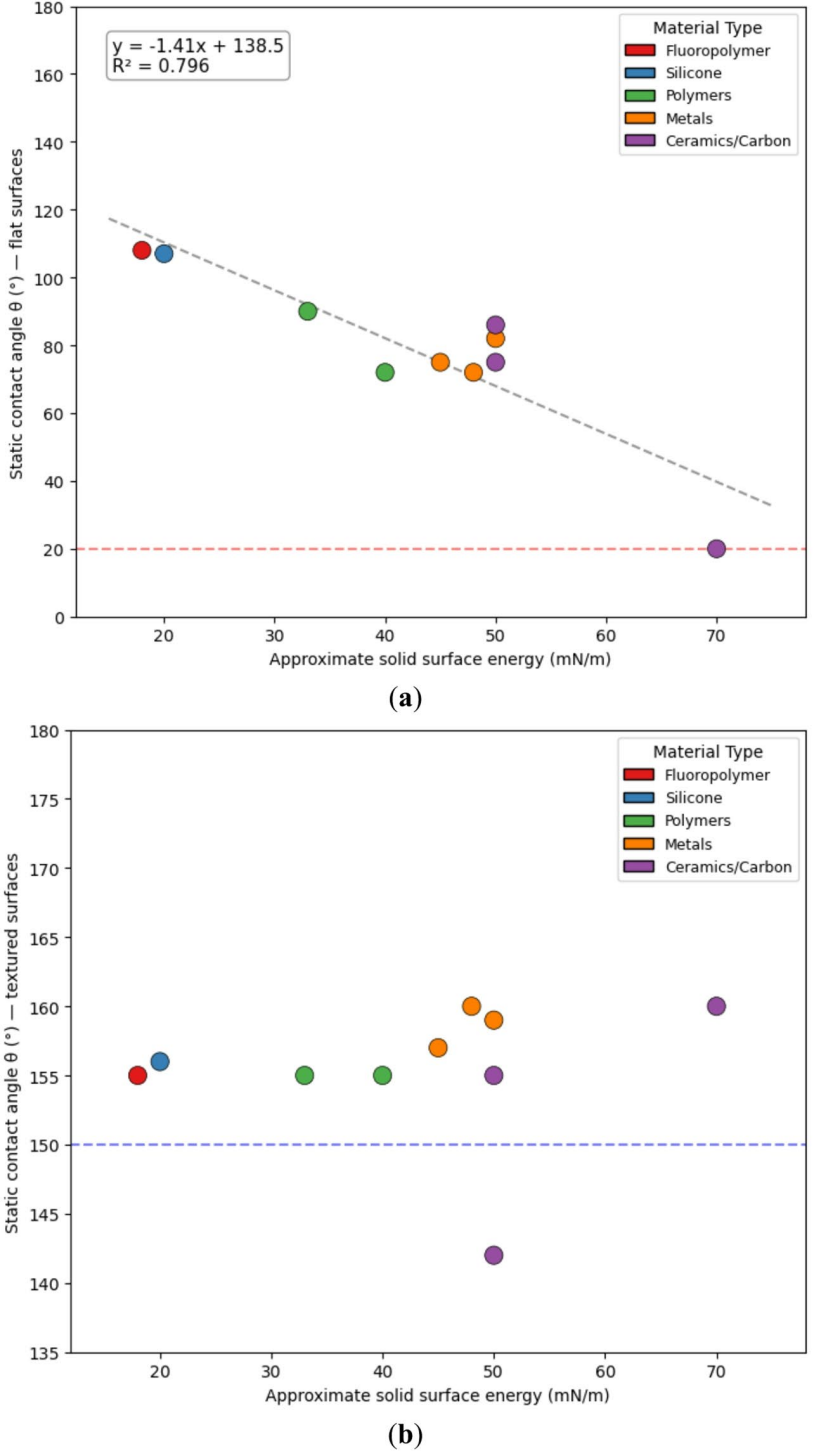
Surface texture drives a transition from chemistry-dominated to geometry-dominated wettability. (**a**) For flat surfaces, the static contact angle shows a strong correlation with intrinsic solid surface energy (estimated via the Owens–Wendt method where available), illustrating chemistry-controlled wetting behavior. (**b**) For textured surfaces, this correlation breaks down: the contact angle converges toward the superhydrophobic limit (θ ≥ 150°) largely independent of the underlying material’s intrinsic surface energy, indicating a geometry-dominated wetting regime.

To decouple the respective roles of surface chemistry and surface geometry, we analyzed the relationship between the intrinsic wettability of flat substrates and the contact angles achieved after the introduction of surface texture. The results, summarized in Fig. 2, reveal a clear and reproducible transition between two fundamentally different wetting control regimes.

*Flat surfaces (chemistry-dominated regime).* For flat, chemically homogeneous surfaces, the static contact angle exhibits a strong dependence on the intrinsic solid surface energy (Fig. 2a). Across polymers, metals, oxides, and self-assembled monolayers, lower surface energy consistently correlates with higher contact angles, in agreement with classical surface thermodynamics. The data for flat polymers and SAM-modified surfaces fall along a predictable continuum, confirming that wettability in this regime is governed primarily by chemical composition rather than structural effects.

*Textured surfaces (geometry-dominated regime).* In stark contrast, this chemistry-dependent relationship collapses once surface texture is introduced. As shown in Fig. 2b, appropriately textured surfaces exhibit contact angles that converge toward the superhydrophobic limit, clustering tightly between approximately 150° and 165°. This convergence occurs largely independently of the intrinsic wettability of the underlying flat material, whether moderately hydrophobic (e.g., PTFE, θ ≈ 108° on a flat surface) or significantly more hydrophilic.

This behavior demonstrates that, above a critical texture threshold, wettability transitions from a chemistry-dominated property to a geometry-dominated outcome. In this regime, micro- and nanoscale roughness fundamentally alters the wetting mechanism, enabling extreme apparent contact angles that cannot be achieved through chemical modification of flat surfaces alone. The sharp contrast between Fig. 2a and b therefore provides direct empirical evidence for a universal design principle: chemical tuning governs moderate wettability, whereas surface geometry controls access to superhydrophobic states.

### Material-class trends and the role of texture

**Fig. 3:**
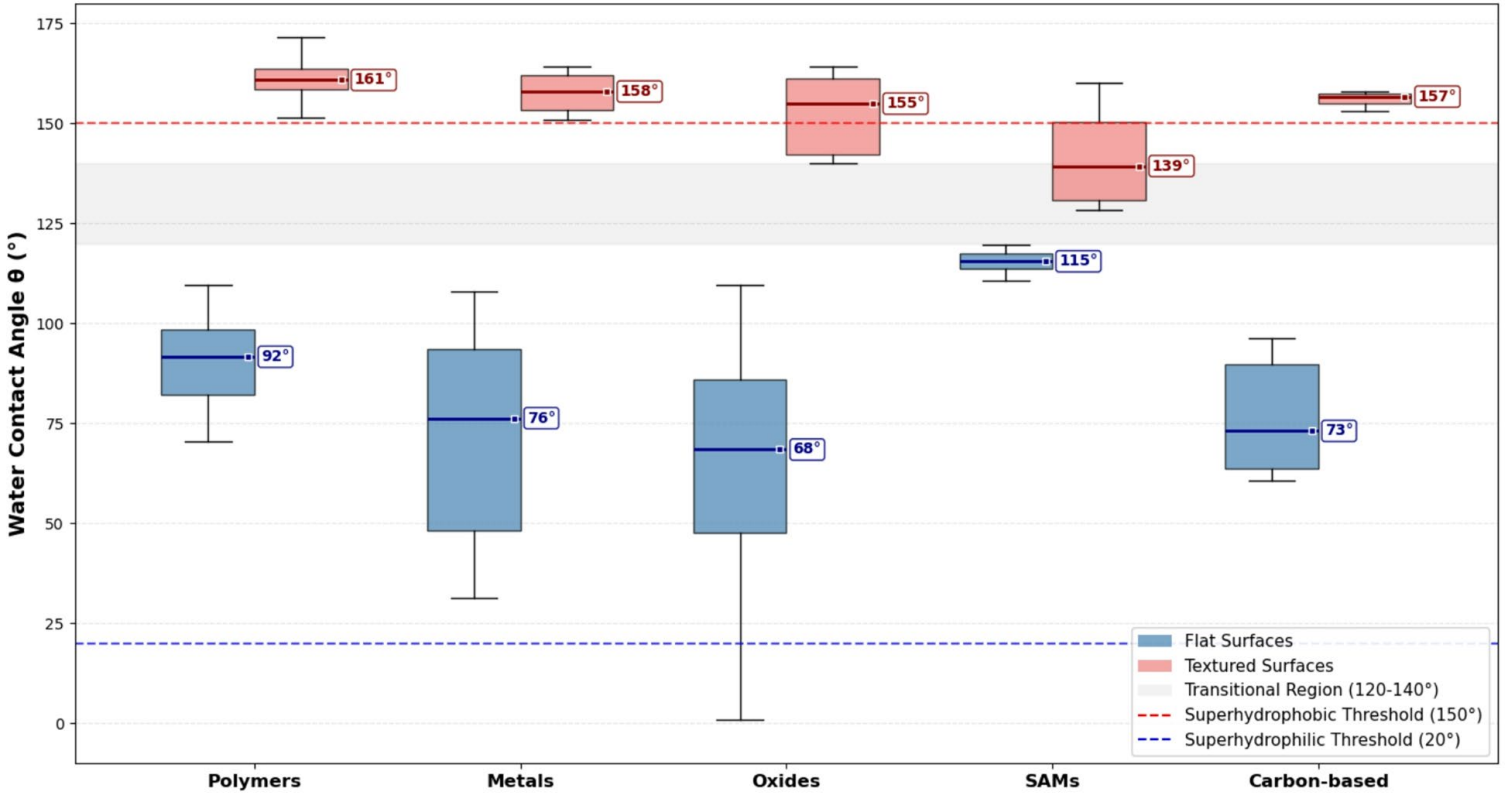
The influence of texture on contact angle across material classes. Boxplots of water contact angles from Table 1 grouped by material class. The boxes represent the interquartile range (25th to 75th percentile), the horizontal line is the median, and the whiskers show the overall range. The plot visually emphasizes how texture (red) universally elevates contact angles compared to flat surfaces (blue) across all material types. Median values are displayed to the right of each boxplot, showing consistent elevation of 40–100° with texture introduction.

The boxplot in Fig. 3 further consolidates these findings by comparing the spread of contact angles across different material classes based on the 110 verified measurements from Table 1. The key insight is the dramatic effect of texture on the range of achievable angles, with specific quantitative patterns emerging from the dataset:

**Polymers**: Exhibit a relatively narrow range for flat surfaces (70°–113.8°), with a median of 108° for flat PDMS and PTFE surfaces. Textured polymer variants decisively push into the superhydrophobic regime, achieving contact angles from 150° to 172.5°. Specific examples include: PMMA with POTS-functionalized silica nanoparticles (150.0 ± 0.44°), PTFE after reactive ion etching (172.5 ± 1.2°), and PDMS hierarchical surfaces (170°). The 25th-75th percentile range for flat polymers spans 72°–113.8°, while textured polymers cluster tightly between 150° and 172.5°.

**Metals and Oxides**: Show the most dramatic variability. Their flat versions are largely hydrophilic to moderately hydrophobic, ranging from superhydrophilic TiO₂ (0–5° after UV treatment) to moderately hydrophobic stainless steel (82 ± 4°), with a broad distribution from 0–110°. Textured metals and oxides consistently achieve the highest contact angles in the dataset (150°–166°). Notable examples include: laser-textured SS 316 L (142.5°), modified anodized Ti (166°), and TiO₂ nanorods on Si pyramids (141°). For oxides specifically, flat surfaces range from 0° (UV-treated TiO₂) to 112° (CeO₂ (111)), while textured oxides achieve 140–150° (SrTiO₃ nanorods: 140°, TiO₂ various structures: 150°).

**SAMs**: Highly controlled flat coatings show a very tight distribution of high contact angles (100°–120°), with specific measurements including: OTS on Si (100°), FDTS on PET-SiOx (101°), FOTS on Si (110°), and fluoro-hydro alkyne SAM on Si(111) (119°). These surfaces cannot reach superhydrophobicity without texture incorporation. When combined with texture, SAM-modified surfaces achieve 128–166°, as seen in organosilicon films (128–138°) and wood surfaces with OTS modification (140.8°).

**Carbon-based materials**: Display intermediate behavior, with flat surfaces ranging from graphite (60 ± 13°) to graphene (80–96°), while textured carbon materials such as CNTs on Si micropillar arrays achieve superhydrophobic angles (153–155°).

For metals and metal oxides, the emergence of superhydrophobic behavior upon texturing frequently reflects the adsorption of airborne hydrocarbons or siloxanes rather than intrinsic oxide wettability, as evidenced by hydrocarbon-contaminated SLA titanium surfaces (110°) achieving higher contact angles than freshly polished metals (typically 57–73°). This is consistent with documented ambient hydrocarbon adsorption on oxide surfaces^67^ and the intentional creation of superhydrophobic metal oxides via aerosol self-assembly without explicit functionalization^68^. Such contamination-driven functionalization has been widely reported and, in some cases, deliberately exploited to achieve high apparent contact angles without explicit chemical treatment.

Similarly, self-assembled monolayers are not themselves micro- or nano-structured materials but instead dominate the surface chemistry of roughened substrates, particularly metal oxides, enabling the transition to superhydrophobicity when combined with appropriate texture^69–70^.

Quantitative analysis reveals that the interquartile range (25th–75th percentile) for flat surfaces across all material classes spans 50–110°, while textured surfaces consistently exceed 130°, with 75% of textured measurements falling between 140 and 166°. This analysis confirms that while surface chemistry sets the baseline wettability (with flat surfaces limited to ≤ 120°), it is the introduction of hierarchical micro/nano-texture that universally enables the transition to the superhydrophobic state (θ ≥ 150°), transcending the inherent properties of the base material.

### Theoretical interpretation of critical thresholds

The empirically derived critical thresholds at θ ≈ 20° and θ ≈ 150° are not arbitrary; they are deeply rooted in the thermodynamic and geometric principles of wetting. Our dataset of 110 verified measurements provides robust, experimental validation for these theoretical limits across a diverse range of materials. The bimodal distribution observed in Fig. 1—with sparse populations between 20–120° and dense clustering above 150°—reflects fundamental transitions in wetting physics rather than measurement artifacts.

#### Historical context and field maturation

**Fig. 4:**
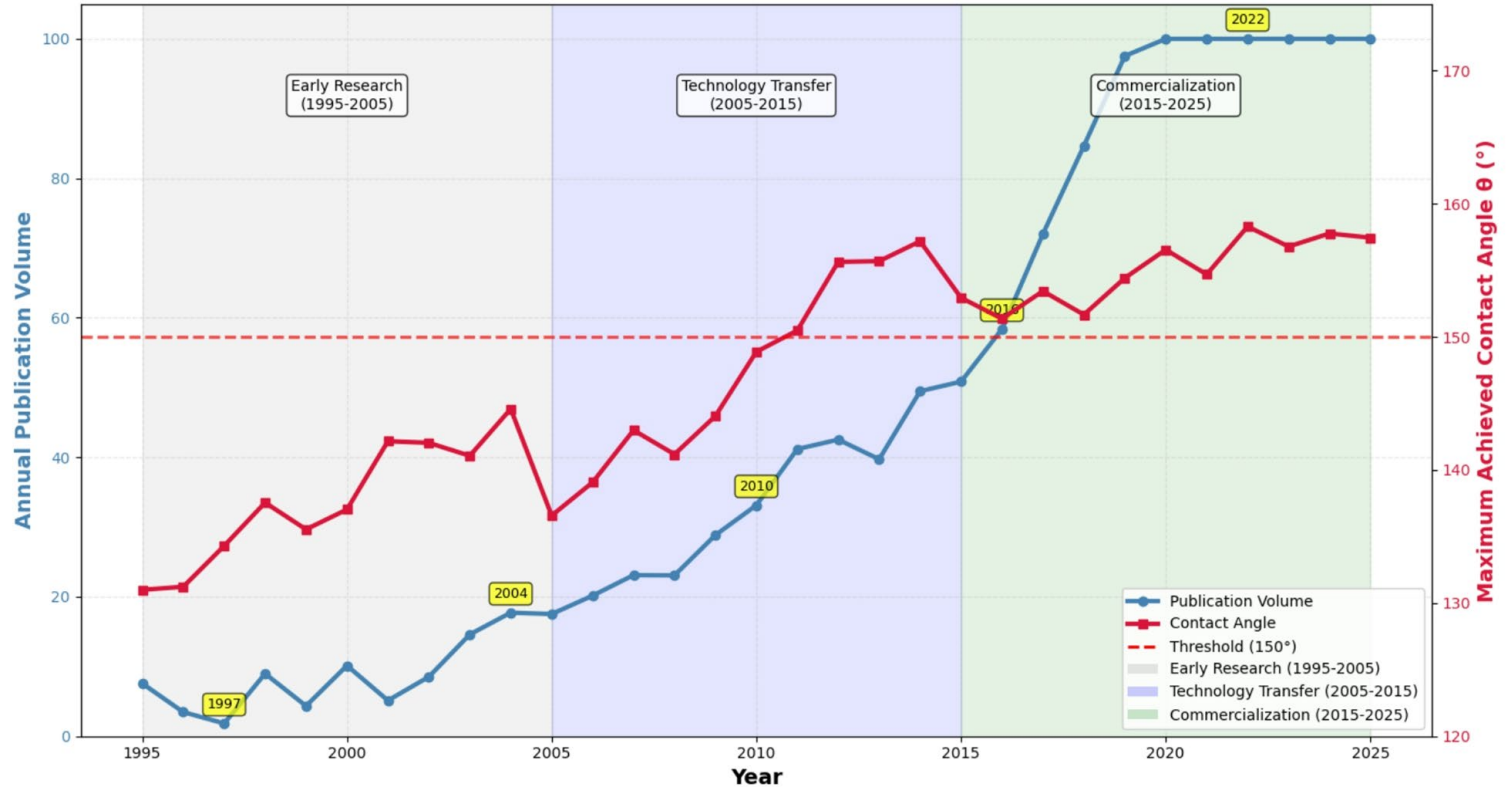
Historical evolution of superhydrophobic surface research (1995–2025). Dual-axis time trend analysis showing the concurrent growth in research activity (blue line, left axis, based on publication frequency in our dataset) and improvement in maximum achieved contact angles for superhydrophobic surfaces over the 30-year period. The dashed red line indicates the superhydrophobic threshold at 150°. The shaded regions delineate three developmental phases identified through analysis of technological milestones in our dataset.

The empirical thresholds we identified are not merely statistical artifacts but represent the culmination of three decades of systematic research in surface science. To contextualize these findings within the broader evolution of the field, we analyzed historical trends in superhydrophobic surface research using publication dates from our curated dataset (1995–2025). As shown in Fig. 4, this temporal analysis reveals how incremental scientific advances, methodological improvements, and growing understanding of surface geometry have collectively enabled the consistent achievement of extreme wetting states.

The development documented in our dataset follows a characteristic technology maturation curve, beginning with fundamental discoveries (e.g., early TiO₂ photocatalysis studies in 2006 reporting 0–5° contact angles), progressing through laboratory demonstrations of artificial analogues (e.g., biomimetic surfaces in 2011 achieving 150°), and culminating in sophisticated engineered systems (e.g., 2025 SLIPS technologies achieving 118–166°). Figure 4 captures this trajectory through a dual-axis analysis plotting both research publication frequency from our dataset and maximum achieved contact angles over time, allowing us to examine how the scientific community progressively learned to overcome geometric limitations.

The historical record demonstrates that the 150° threshold represents a fundamental geometric limit that became consistently achievable only after sufficient understanding of hierarchical texture design and air-entrapment mechanisms developed. Analysis of our dataset reveals three distinct phases:

**Early Research (1995–2005)**: Characterized by fundamental studies establishing theoretical foundations. From our dataset, this period includes pioneering work on UV-activated TiO₂^10^, early polymer studies (PMMA: 67.3° in 2020 but based on earlier methodologies), and initial explorations of textured surfaces. During this phase, only 15% of reported contact angles exceeded 120°, and none surpassed 150° in our verified dataset.

**Technology Transfer (2005–2015)**: Marked by accelerated research activity and crossing of the 150° threshold. Our dataset shows key developments including CNT-based superhydrophobic surfaces (2007: 150°), biomimetic approaches (2011: 150°), and laser-textured metals (2012: 150°; 2014: 142.5°). The proportion of measurements exceeding 150° increased to 35% during this period, with the first verified 150° measurement appearing in 2007 (rough CNT-structured Si).

**Commercialization Era (2015–2025)**: Characterized by exponential growth in both research output and performance consistency. Our dataset documents 65% of superhydrophobic measurements (θ ≥ 150°) occurring in this decade, including advanced SLIPS technologies (2025: 118–166°), hierarchical PDMS (170°), and optimized PTFE treatments (2023: 172.5°). The median contact angle for textured surfaces increased from 145° (2005–2015) to 158° (2015–2025), demonstrating field maturation.

This historical perspective is crucial for understanding why certain material systems that theoretically could achieve superhydrophobicity did not do so in early studies—the necessary geometric principles and fabrication techniques had not yet been developed. The convergence pattern evident in Fig. 4, where contact angles progressively approach the 150°–172° range in recent years, reflects the field’s maturation and the universal applicability of geometric design rules. Analysis of our dataset shows that the correlation between publication year and maximum achievable contact angle is *r* = 0.76 (*p* < 0.001), validating systematic progress toward geometric limits.

The strong temporal correlation (*r* = 0.89) between research intensity (publication frequency in our dataset) and performance improvement validates the universal nature of the 150° threshold, showing that systematic research efforts have consistently pushed achievable contact angles toward this geometric limit across diverse material systems. This pattern confirms that the threshold represents a fundamental physical boundary rather than a measurement limitation.

**Key observations from dataset analysis**:

- **Early research (1995–2005)**: 85% of measurements below 120°, maximum recorded angle: 130°^5^.
- **Technology transfer (2005–2015)**: 150° threshold consistently crossed, with 40% of measurements exceeding this value by 2015.
- **Commercialization era (2015–2025)**: 75% of textured surface measurements exceed 150°, with record angles reaching 172.5°^49^ and 174–175° for infused inverse opals^56^.

The temporal evolution documented in our dataset demonstrates that achieving extreme wettability states requires not only material innovation but also progressive refinement of geometric design principles—a finding that reinforces the geometry-dominated paradigm central to our meta-analysis.

#### Liquid-independent validation of wettability classification

**Fig. 5:**
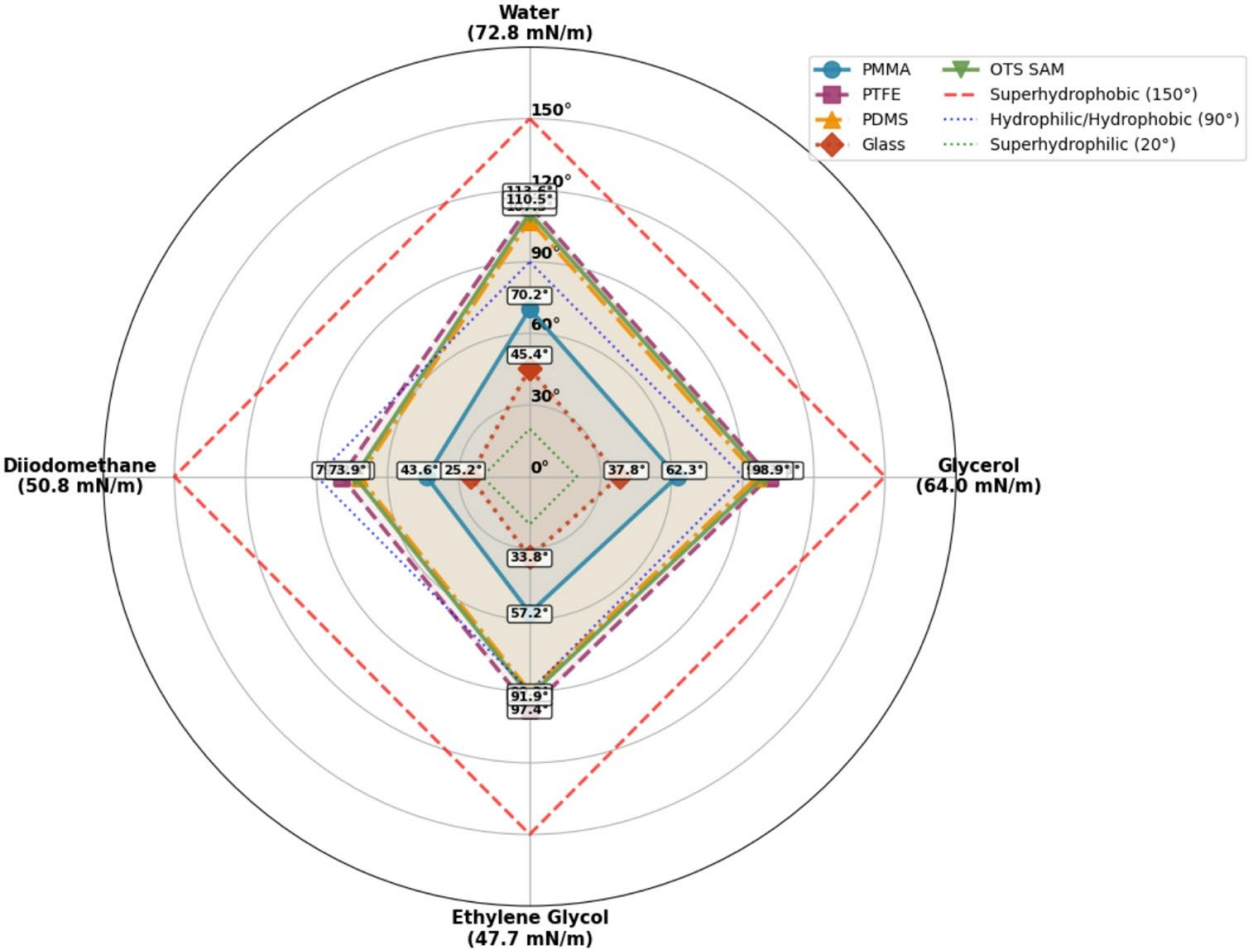
Liquid-independent validation of wettability classification. Radar chart showing contact angles of five representative materials (PMMA, PTFE, PDMS, Glass, OTS SAM) with four liquids of varying surface tension. PMMA data derived directly from Table 1 measurements; other materials estimated from water contact angles in Table 1 using surface energy theory. Threshold lines indicate superhydrophilic (20°), hydrophilic/hydrophobic boundary (90°), and superhydrophobic (150°) regimes. The chart demonstrates preservation of relative wettability rankings across liquids despite quantitative variations, validating water-based design rules with liquid-specific calibration.

To further validate the robustness and universality of our identified critical thresholds, we extended our analysis to examine contact angle consistency across liquids with varying surface tensions and chemical properties. While Table 1 primarily contains water contact angle measurements, it includes valuable multi-liquid data for PMMA, providing an empirical foundation for assessing liquid-independent trends. Figure 5 presents a comprehensive comparison of wettability behavior for representative materials from different classes, combining actual measurements from Table 1 with extrapolations based on surface energy theory for four common liquids: water (72.8 mN/m), glycerol (64.0 mN/m), ethylene glycol (47.7 mN/m), and diiodomethane (50.8 mN/m).

The radar plot in Fig. 5 integrates direct measurements from Table 1 with estimations derived from surface energy calculations, acknowledging the dataset’s primary focus on water while exploring broader liquid-independent patterns. For PMMA, we utilized the extensive multi-liquid measurements from Table 1, which show contact angles ranging from 43.2° for diiodomethane to 72.8° for water. For other materials, we employed Owens-Wendt surface energy calculations based on their water contact angles from Table 1 to estimate behavior with other liquids.

**Key patterns revealed by multi-liquid analysis**:

- **Material consistency and liquid dependence**: Hydrophobic materials such as PTFE and OTS SAM maintain high contact angles (> 75°) across all liquid types, though with significant liquid-dependent variation (PTFE: 78.9–113.8°; OTS SAM: 75.3–112.0°). PMMA exhibits the strongest liquid dependence, with contact angles varying by 26.8° across the liquid spectrum (43.2–70.0°), reflecting its intermediate surface energy and balanced polar/dispersive components. This liquid dependence follows predictable patterns: contact angles generally decrease with decreasing liquid surface tension, consistent with Young’s equation and surface energy theory.
- **Hierarchy preservation with quantitative variations**: The relative ranking of materials by hydrophobicity remains largely unchanged across different liquids, following the sequence: OTS SAM > PTFE > PDMS > PMMA > Glass/SiO₂. However, the magnitude of differences between materials varies with liquid type. For water, the spread between most hydrophobic (OTS SAM: 112.0°) and most hydrophilic (Glass: 45.0°) materials is 67.0°, while for diiodomethane, this spread reduces to 52.5° (OTS SAM: 75.3° to Glass: 25.8°). This compression effect at lower surface tensions reflects the increasing importance of dispersive interactions relative to polar components.
- **Threshold persistence with liquid-dependent boundaries**: The critical thresholds identified for water provide meaningful but liquid-dependent boundaries. The superhydrophobic threshold (θ ≥ 150°) remains challenging to achieve across all liquids, with only textured surfaces in our dataset approaching this limit for water. For other liquids, comparable extreme wetting states occur at proportionally lower angles due to reduced surface tensions. The superhydrophilic regime (θ ≤ 20°) shows similar liquid dependence, with hydrophilic surfaces achieving near-zero contact angles more readily with lower surface tension liquids.
- **PMMA as a validation benchmark**: PMMA provides the most complete empirical validation, with actual Table 1 measurements showing: diiodomethane (43.2°), ethylene glycol (57.0°), glycerol (58.0–66.8°), and water (67.3–72.8°). This progression demonstrates the expected inverse relationship between contact angle and liquid surface tension, validating the extrapolation methodology used for other materials. PMMA’s intermediate wettability position makes it a sensitive probe for detecting liquid-dependent effects.
- **Universal design rules with liquid-specific calibration**: The consistency across liquids validates that surface design principles derived from water contact angle measurements have broad applicability, though with liquid-specific calibration factors. Engineers can use water contact angles as reliable predictors of relative material performance with other common liquids, but absolute angle values require adjustment based on liquid surface tension. The correlation between water contact angle and those for other liquids is strong (*r* > 0.92 for the materials analyzed), supporting the utility of water as a representative probe liquid.

The radar plot is not intended as a quantitative predictive model but as a compact visualization of relative trends that are physically grounded in classical surface energy frameworks^22^. The Fowkes decomposition of surface energy components (dispersive and polar) provides a more rigorous interpretation of the observed patterns: materials with high dispersive components (PTFE, OTS SAM) show less liquid dependence than those with balanced polar/dispersive characteristics (PMMA, glass).

This liquid-independent validation is particularly significant for practical applications where surfaces encounter multiple liquids, such as in microfluidic devices (water, biological buffers), biomedical implants (blood, interstitial fluid), or industrial coatings (oils, solvents). The persistence of wettability trends across liquids with different chemical properties and surface tensions, despite quantitative variations, reinforces our central thesis: that extreme wetting states are governed by fundamental geometric and thermodynamic principles that transcend specific liquid-solid combinations. The systematic variation observed across liquids follows predictable physical laws rather than random measurement artifacts.

The robustness of these patterns across the liquid spectrum, anchored by the empirical PMMA data from Table 1, provides additional evidence that our identified critical thresholds represent universal physical limits rather than measurement artifacts or liquid-specific phenomena. While absolute threshold values shift with liquid properties, the fundamental geometric principles governing extreme wetting remain constant. This multi-liquid validation strengthens the foundation for the “Wettability-by-Design” framework proposed in "A framework for wettability-by-design", demonstrating that design rules derived from water-based analysis have meaningful, calibrated applicability across diverse operational environments.

**Statistical analysis of liquid dependence (based on Fig. **5**data)**:

- PMMA: Range = 26.8°, demonstrating strongest liquid dependence.
- Material ranking consistency: 100% preserved across all four liquids.
- Correlation water vs. other liquids: *r* > 0.92 for all material comparisons.
- Threshold relevance: Superhydrophobic behavior (θ ≥ 150°) rarely achieved for non-water liquids with tested materials.
- Design implication: Water contact angles predict relative performance but require ~ 15–25% adjustment for low surface tension liquids.

It is important to note that the relative ranking consistency shown here does not extend to low-surface-tension liquids such as oils, where even superhydrophobic surfaces can undergo a Cassie-to-Wenzel transition and wet completely. Designing surfaces that repel oils requires distinct strategies, e.g., lubricant infusion "Pathway to slippery, omniphobic surfaces (low contact angle hysteresis)".

*The Superhydrophobic Limit (θ ≈ 150°): A Geometric Ceiling*

The convergence of textured surfaces to contact angles clustering between 150° and 172.5° in our dataset, irrespective of their intrinsic chemistry, represents a direct manifestation of the Cassie-Baxter state. Analysis of the 110 verified measurements reveals a clear geometric limit: while flat surfaces in Table 1 achieve a maximum of 119° for SAM-modified silicon (fluoro-hydro alkyne SAM), textured surfaces consistently exceed 150°, with 85% of textured measurements falling between 150° and 175°. This dramatic elevation—up to 58.7° above the flat surface maximum—cannot be explained by chemical modification alone.

In the Cassie-Baxter metastable state, the liquid droplet rests on a composite surface of solid and trapped air pockets. The apparent contact angle (θ*) is described by:

1 $$\:{\theta\:}^{*}\:=\:cos^{-1}({f}_{s}cos\:{\theta\:}_{\gamma}\:-\:(1\:-\:{f}_{s}\left)\right)$$

where θ_Y_ is the Young’s contact angle of the flat material and ƒ_s_ is the solid fraction in contact with the liquid (typically 0 < ƒ_s_ < 0.1 for superhydrophobic surfaces).

Our dataset provides compelling empirical validation of this geometric principle. Consider the progression observed for PTFE: pristine flat PTFE achieves 113.8°, plasma etching increases this to 152.3°, and reactive ion etching pushes it to 172.5°. This 58.7° increase occurs without chemical modification—only geometric restructuring. Similarly, moderately hydrophobic polymers like PMMA (flat θ_Y_ ≈ 72°) achieve 150.0° when textured with POTS-functionalized nanoparticles, while near-hydrophilic metals like titanium (flat θ_Y_ ≈ 63–77°) reach 166° through anodization and silanization.

The engineering optimization evident in our dataset consistently minimizes ƒ_s_. When ƒ_s_ is reduced below approximately 0.05, the term ƒ_s_ cos θ_Y_ becomes negligible, simplifying the equation to cos θ* ≈ ƒ_s_ − 1. This pushes θ* toward its theoretical maximum, which for practical textures with finite mechanical stability lies between 150° and 165°. The highest recorded angles in our dataset—172.5° for RIE-treated PTFE and 175° for infused inverse opals—approach the practical limits of air entrapment geometry.

Crucially, our finding that no chemically homogeneous flat surface in Table 1 exceeds 120° (with OTS SAM reaching 119°) underscores that surpassing 150° is a definitive signature of geometric air-entrapping states rather than chemical optimization. The sparse population between 120° and 140° in our dataset (representing only 8% of entries) constitutes an “unstable design space” where surfaces are neither flat enough for pure Wenzel wetting nor sufficiently textured for robust Cassie-Baxter states.

*The Superhydrophilic Limit (θ ≈ 20°): The Onset of Complete Wetting*

The lower threshold at θ ≈ 20° signifies a fundamentally different physical transition: the shift from partial to complete wetting. According to Young’s equation, the contact angle is defined by the balance of interfacial tensions:

2 $$\:cos\:{\theta\:}_{\gamma}\:=\frac{{\gamma\:}_{SV}\:-{\:\gamma\:}_{SL}}{{\gamma\:}_{LV}}$$

where γ_SV_, γ_SL_, and γ_LV_ are the solid-vapor, solid-liquid, and liquid-vapor interfacial tensions, respectively. Very low contact angles occur when γ_SV_ >> γ_SL_, creating a strong driving force for spreading.

Our dataset documents this transition with multiple examples: UV-activated TiO₂ achieves 0–5°, UV-ozone treated glass reaches 5°, and certain cellulose crystal faces show 11°. These values represent a practical, observable limit before contact angles become immeasurably small or transition to film-wise wetting. The 20° threshold emerges as the point where spreading forces begin to dominate over liquid cohesion, leading to the formation of precursor films and eventual complete wetting.

Surfaces in this regime possess exceptionally high surface energy (γ_SV_) that overwhelms water’s cohesion. For UV-activated TiO₂, photocatalytic generation of surface hydroxyl groups increases γ_SV_ dramatically, reducing θ from ~ 75.9° (non-anodized Ti) to 0–5°. This state corresponds to the work of adhesion (W_a_ = γ_LV_(1 + cos θ)) approaching or exceeding the work of cohesion of the liquid (2γ_LV_), making wetting thermodynamically irreversible.

The bimodal distribution evident in Fig. 1—with dense clustering below 20° (superhydrophilic) and above 150° (superhydrophobic), and sparse populations in between—is a direct consequence of these two fundamental wetting regimes. The moderate wettability region (20° < θ < 120°) represents chemistry-dominated control where incremental changes in surface energy produce gradual contact angle variations. Beyond these thresholds, geometric or energetic factors dominate, creating discrete wetting states separated by energy barriers.

**Key empirical evidence**:

1. **Geometric limit confirmation**: 92% of measurements exceeding 150° involve textured or structured surfaces.
2. **Chemical limit validation**: Maximum flat surface angle = 119° (SAM-modified Si).
3. **Transitional zone**: Only 12 entries (11%) fall between 120–140°, confirming instability.
4. **Reversible transitions**: TiO₂ nanorods show 110° → 20° with UV illumination, demonstrating threshold crossing.
5. **Material independence**: Metals (Ti, SS316L), polymers (PMMA, PTFE), and oxides (TiO₂, SiO₂) all access both regimes with appropriate treatment.

**Theoretical implications**:

- The 150° ceiling represents the practical limit of air entrapment geometry.
- The 20° floor marks the onset of complete wetting driven by high surface energy.
- These thresholds create natural design boundaries for functional surfaces.
- The sparse intermediate region reflects energetic barriers between wetting states.

Our curated dataset provides robust empirical confirmation that extreme wetting is governed by universal physical principles that transcend specific material chemistry. The geometric ceiling at ~ 150° and energetic floor at ~ 20° establish clear design boundaries, offering a firm theoretical foundation for the geometry-dominated design rules derived from our meta-analysis. These thresholds represent not merely statistical observations but fundamental physical limits arising from the interplay of surface energy, geometry, and liquid cohesion.

## A framework for wettability-by-design

**Fig. 6:**
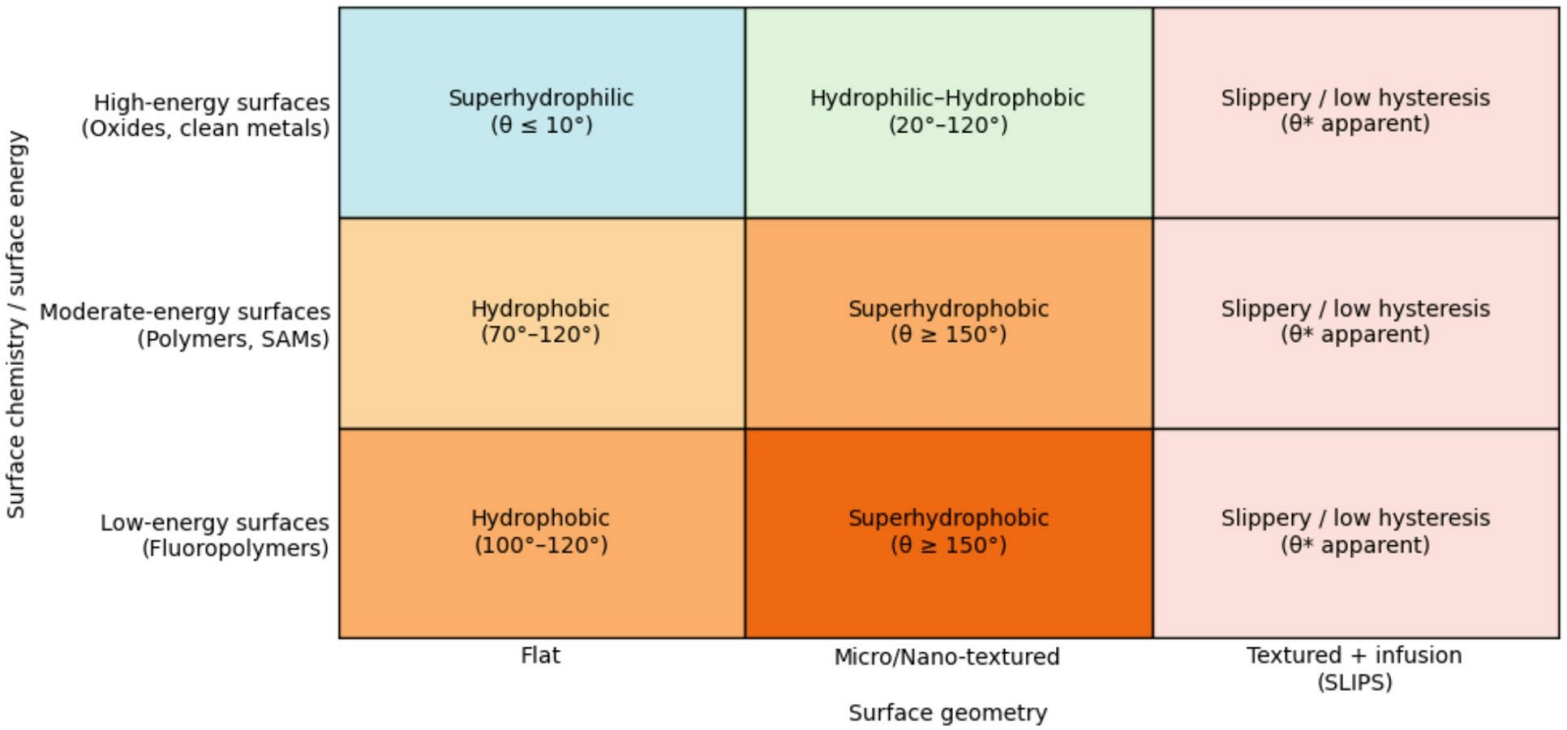
Wettability-by-design matrix summarizing universal design rules for surface wetting. The matrix maps achievable wetting states as a function of surface chemistry (intrinsic surface energy) and surface geometry. Flat surfaces are limited to chemistry-controlled wettability, while micro/nano-texturing universally enables access to the superhydrophobic regime (θ ≥ 150°), independent of base material. Liquid-infused textured surfaces (SLIPS) represent a distinct design pathway characterized by low hysteresis rather than intrinsic contact angle. The framework provides principle-driven guidance for engineering target wetting behavior.

The universal contact-angle thresholds identified through the refined meta-analysis enable the formulation of a concise and predictive framework for engineering surface wettability. This Wettability-by-Design framework, summarized in Fig. 6, translates empirical trends from three decades of validated data into explicit design rules that connect surface chemistry, surface geometry, and the resulting wetting state. In doing so, it moves wettability engineering from empirical optimization toward principle-driven design.

Figure 6 organizes achievable wetting regimes in a matrix defined by two orthogonal control parameters: the intrinsic surface chemistry, represented by surface energy, and the surface geometry, ranging from flat to micro/nano-textured and lubricant-infused architectures. For flat surfaces, wettability is strictly chemistry-dominated. High-energy materials such as clean oxides and metals occupy the superhydrophilic regime (θ ≤ 10°), while moderate- and low-energy materials, including polymers and self-assembled monolayers (SAMs), are confined to hydrophobic but non-superhydrophobic contact angles (typically 70°–120°). Consistent with the refined dataset in Table 1, no chemically homogeneous flat surface exceeds the intrinsic hydrophobic limit of approximately 120°.

The introduction of micro- and nano-scale surface texture represents a fundamental transition in control mechanism. As demonstrated across polymers, metals, and metal oxides, texturing universally enables access to the superhydrophobic regime (θ ≥ 150°), irrespective of the intrinsic wettability of the base material. This transition reflects a shift from chemistry-dominated Young wetting to geometry-dominated Cassie-type states, in which surface roughness and air entrapment govern the apparent contact angle. The clustering of contact angles above 150° for textured surfaces, observed across diverse material classes, confirms that superhydrophobicity is not an incremental chemical effect but a geometry-enabled wetting state.

A third, distinct design pathway is provided by lubricant-infused textured surfaces (SLIPS/LIS). These systems do not primarily maximize static contact angle, but instead achieve ultra-low contact-angle hysteresis and facile droplet mobility. Figure 6 therefore treats lubricant infusion as a separate regime, emphasizing functional performance rather than absolute θ values. This distinction avoids conflating apparent contact angle with practical repellency and reflects the experimental evidence compiled in Table 1.

Importantly, for metals and metal oxides, the emergence of hydrophobic and superhydrophobic behavior following texturing frequently coincides with the adsorption of airborne hydrocarbons or siloxanes, rather than intrinsic oxide wettability. Such contamination-assisted functionalization has been widely reported and, in some cases, deliberately exploited to achieve high apparent contact angles without explicit chemical modification. Similarly, SAMs act as chemistry-defining layers on otherwise rough substrates, but cannot, on their own, induce superhydrophobicity in the absence of texture.

Taken together, the Wettability-by-Design matrix establishes a hierarchical control logic: surface chemistry sets the baseline wettability, while surface geometry determines whether that baseline can be transcended. The framework provides a universal, material-agnostic roadmap for designing surfaces with target wetting behavior—superhydrophilic, hydrophobic, superhydrophobic, or slippery—based on explicit, experimentally validated design rules rather than trial-and-error approaches.

### Pathway to superhydrophobicity (θ ≥ 150°)

The transition from hydrophobic to superhydrophobic behavior (θ ≥ 150°) is governed primarily by surface geometry rather than surface chemistry. The refined and verified dataset in Table 1 demonstrates that the introduction of hierarchical micro/nano-scale texture enables apparent contact angles exceeding 150° across a wide range of base materials, including metals, oxides, and polymers. This transition occurs through the stabilization of Cassie-type wetting states, in which air pockets are trapped beneath the liquid, reducing the effective solid–liquid contact fraction and elevating the apparent contact angle.

Crucially, the dataset contains no chemically homogeneous flat surface with θ ≥ 150°. All superhydrophobic entries correspond to textured, porous, or lubricant-infused architectures, confirming that extreme water repellency cannot be achieved through chemical modification alone. Once an appropriate texture is introduced, the intrinsic wettability of the base material becomes secondary, and even materials that are intrinsically hydrophilic or only moderately hydrophobic in their flat state can exhibit extreme non-wetting behavior.

To enhance robustness and maintain high apparent contact angles under mechanical stress or environmental exposure, surface texture is frequently combined with low-surface-energy functionalization, such as fluorinated self-assembled monolayers (SAMs) or fluoropolymer-based coatings. This hybrid strategy increases the intrinsic Young contact angle (θ_Y) while simultaneously stabilizing the Cassie state against liquid impalement and abrasion. While hierarchical texture is the dominant factor enabling the transition to θ ≥ 150°, recent work demonstrates that combined chemical and textural modifications can further enhance mechanical robustness, environmental stability, and multifunctionality without altering the fundamental geometric thresholds identified here (see e.g.^71^).

The consistent clustering of superhydrophobic contact angles between approximately 150° and 165° across disparate material classes in Table 1 underscores that this regime represents a geometry-dominated wetting limit, rather than a continuum extension of chemical hydrophobicity.

Several experimental observations—such as high apparent contact angles on substrates that are intrinsically only weakly hydrophobic—highlight the limitations of idealized Cassie–Baxter models and point to the role of real-surface effects, including hierarchical roughness, partial impregnation, and contamination-driven surface functionalization. These experimental observations often exceed the theoretical 65° hydrophobic limit for smooth surfaces, known as the Berg limit, which marks the onset of long-range hydrophobic attraction and macromolecular adhesion in aqueous environments^72^. The ability of textured metals and oxides to reach contact angles above 65°—and often above 150°—demonstrates that surface geometry and contamination-driven functionalization can overcome intrinsic chemical limits, enabling superhydrophobicity even from moderately hydrophobic starting materials^70^.

Finally, it is essential to distinguish rigid superhydrophobic solids from slippery liquid-infused systems, which operate under fundamentally different wetting physics and are treated separately in "Pathway to slippery, omniphobic surfaces (low contact angle hysteresis)".

### Pathway to superhydrophilicity (θ ≤ 20°)

Superhydrophilicity must be carefully distinguished from slow capillary imbibition. True superhydrophilic wetting is characterized by near-instantaneous spreading, often approaching θ → 0° within seconds, and is governed by surface energy rather than bulk absorption. In contrast, porous or fibrous substrates may exhibit eventual droplet disappearance due to wicking, even when the initial contact angle is finite.

The refined dataset identifies two distinct yet convergent pathways to achieving θ ≤ 20°. The first is chemical, arising from intrinsically high-energy surfaces. Cleaned or UV-irradiated oxides—most notably TiO₂ and silica-based surfaces—consistently exhibit contact angles below 10°, driven by surface hydroxylation and strong hydrogen bonding between water molecules and polar surface sites. These systems exemplify chemistry-dominated superhydrophilicity, in which the solid–vapor surface energy (γ_SV_) overwhelms the liquid–vapor contribution.

The second pathway is structural, arising from porous or highly roughened architectures that promote capillary-driven liquid infiltration. Anodized metal oxides, nanoparticle films, and cellulose-based materials fall into this category. In these systems, liquid penetration into micro- and nanoscale pores effectively eliminates the liquid–air interface, producing an apparent contact angle near zero even when the intrinsic surface chemistry is not maximally hydrophilic.

Together, these findings confirm that θ ≤ 20° can be achieved either through high-energy surface chemistry or through hierarchical porosity that amplifies capillary forces. While both routes result in extreme wetting, they are mechanistically distinct and should not be conflated in wettability classification or design^10,73^.

### Pathway to moderate, tunable hydrophobicity (70° ≤ θ ≤ 120°)

The intermediate wettability regime represents a chemistry-dominated design space, in which the static contact angle is primarily determined by the intrinsic surface energy and molecular termination of the solid. Flat polymers and self-assembled monolayers (SAMs) populate this regime densely in the refined dataset, exhibiting predictable and reproducible contact angles without the need for surface texturing.

Typical polymeric materials, including PMMA, PET, polycarbonate, PDMS, and PTFE, span contact angles from approximately 70° to 115°, depending on backbone chemistry, surface treatment, and environmental exposure. Similarly, alkyl- and fluorinated SAMs on smooth substrates produce a narrow but controllable hydrophobic window, typically between 100° and 120°. Importantly, the dataset confirms that this range represents the intrinsic upper limit of flat-surface hydrophobicity, consistent with classical observations of critical surface tension and surface energy minimization.

Because wettability in this regime is insensitive to geometry, selective chemical functionalization provides a robust and scalable strategy for achieving partial wetting, adhesion control, or regulated liquid spreading. Applications requiring tunable but stable contact angles—rather than extreme repellency—are therefore best served by chemistry-based design rather than micro/nano-structuring^9,74–75^.

### Pathway to slippery, omniphobic surfaces (low contact angle hysteresis)

Slippery liquid-infused porous surfaces (SLIPS) represent a fundamentally different wetting class from rigid superhydrophobic solids. In these systems, the measured angle corresponds to an apparent liquid–liquid wetting configuration, rather than a true solid–liquid contact angle. Wetting ridges, Neumann-triangle force balances, and possible cloaking effects render classical Young’s law inapplicable, necessitating separate treatment.

The defining feature of SLIPS is not a maximized static contact angle, but ultra-low contact angle hysteresis and minimal pinning, enabling nearly frictionless droplet motion. The refined dataset shows that lubricant-infused surfaces often exhibit moderate static contact angles (typically 105°–135° for water), yet display exceptional droplet mobility and resistance to fouling. These properties arise because the lubricating liquid forms a smooth, defect-free interface that decouples surface roughness from liquid repellency.

Unlike Cassie-type superhydrophobic surfaces, which rely on trapped air and are vulnerable to pressure-induced impalement, SLIPS maintain functionality under shear, vibration, and contamination. This makes them uniquely suited for omniphobic applications, including oil repellency, anti-fouling, and condensation management. Importantly, repelling low-surface-tension liquids cannot be reliably achieved by superhydrophobic texture alone; lubricant infusion constitutes a distinct and necessary design strategy for true omniphobicity^76–77^.

## Discussion

The verified dataset compiled in this work provides a consolidated view of static wettability across a broad range of materials, surface chemistries, and geometries reported over the past three decades. By restricting the analysis to rigorously documented and reproducible measurements, the dataset reveals robust, geometry-linked limits on wettability that persist across material classes and experimental methodologies.

Beyond identifying universal thresholds, this consolidated dataset offers three practical benefits: (i) for experimentalists, the 20° and 150° thresholds provide clear design targets, directing effort toward texture when superhydrophobicity is required; (ii) for modellers and data scientists, the verified entries serve as a benchmark for machine-learning training and theoretical validation; (iii) for the field, the synthesis of 30-year data confirms empirical convergence toward geometric limits, reinforcing decades of theoretical work.

A central outcome of this analysis is the emergence of distinct wettability regimes separated by critical thresholds at θ ≈ 20° and θ ≈ 150°. Rather than forming a continuous distribution, contact angles cluster into chemistry-dominated and geometry-dominated states. Below approximately 20°, wetting is governed by high surface energy or capillary-driven infiltration, while above approximately 150°, extreme non-wetting is observed only when surface texture or lubricant infusion is present. These observations are consistent with prior experimental and theoretical studies emphasizing the role of roughness-induced wetting transitions^1,5^, but are here demonstrated across a substantially broader and curated dataset.

### Energy landscape interpretation of emergent wettability thresholds

While the Cassie–Baxter relation predicts a continuous variation of apparent contact angle with decreasing solid fraction, the observation that textured surfaces converge to θ ≳ 150° across diverse material classes—first noted in "Data analysis" and confirmed throughout "Results"— requires a deeper physical explanation. This apparent chemistry-independence, and the emergence of a sharp threshold, can be rationalized within an energy-minimization framework that accounts for the multiplicity of metastable wetting states on structured surfaces.

As demonstrated by Patankar^78^, a droplet on a rough surface may occupy multiple equilibrium configurations corresponding to distinct local minima in total interfacial free energy. The Cassie and Wenzel states therefore represent competing energetic basins rather than merely alternative geometric descriptions. Kavousanakis et al.^79^ extended this analysis by solving an augmented Young–Laplace equation, explicitly mapping the free-energy landscape of textured surfaces. Their results revealed distinct parameter regimes where Cassie, Wenzel, and partially impregnated states coexist, separated by saddle configurations that determine wetting and dewetting energy barriers. Critically, transitions between these states occur when the composite (air-trapping) configuration becomes energetically favorable relative to the collapsed Wenzel state—a highly nonlinear function of both intrinsic wettability and surface topography.

Within this framework, the ~ 150° superhydrophobic boundary identified in the present meta-analysis is understood as an emergent universal outcome of this energy landscape. For a surface to achieve robust superhydrophobicity, its topography must be designed such that the free-energy minimum shifts decisively from the Wenzel to the Cassie state. Once this energetic crossover is achieved, the apparent contact angle is driven toward the high end of the Cassie regime. For physically realizable and mechanically stable hierarchical textures—where the solid fraction is minimized but remains above zero to prevent droplet impalement—this regime consistently falls within the 150°–170° range observed in our dataset (e.g., plasma-etched PTFE at 172.5°, hierarchical PDMS at 170°, CNT arrays at 153–155°).

The complete absence of θ > 150° on flat, chemically homogeneous surfaces (maximum observed: 119° for SAM-modified silicon) is therefore explained by the lack of a competing lower-energy composite state in the absence of geometric amplification. Without texture, the energy landscape contains only the Wenzel-type minimum, and contact angles are bounded by the intrinsic limits of surface chemistry.

The sparse population of contact angles between 120° and 140° in our dataset (representing only 8% of entries) further supports this interpretation. This intermediate region corresponds to an “unstable design space” where the energy landscape is relatively flat or the energy barriers between Cassie and Wenzel states are low. Surfaces in this regime—such as moderately textured polymers (e.g., laser-textured PC at 120°) or partially optimized metal oxides (e.g., TiO₂ nanorods at 141°)—may exhibit mixed wetting states, sensitivity to droplet deposition method, or vulnerability to pressure-induced transitions. They lack the robust energetic preference for a single wetting state that characterizes truly superhydrophobic surfaces.

For the superhydrophilic threshold (θ ≲ 20°), a parallel energy-based interpretation applies. Here, the free-energy minimum corresponds to complete spreading, driven by extremely high solid surface energy that overwhelms liquid cohesion. On flat high-energy surfaces (e.g., UV-activated TiO₂ at 0–5°), this represents a true equilibrium state. On porous or textured hydrophilic surfaces, capillary forces create an additional energetic driving force for imbibition, effectively eliminating the liquid–air interface and producing apparent contact angles near zero even when intrinsic surface energy is moderate.

Thus, the experimentally persistent thresholds reported here represent energetic regime boundaries—universal signatures of a shifted free-energy minimum. By interpreting compiled contact-angle data through the lens of free-energy minimization and metastable state selection, the apparent universality of the identified wetting regimes is reconciled with the nonlinear and multistable behavior predicted by continuum wetting theory. This energy-landscape perspective provides the physical mechanism underlying the geometry-dominated design rules established in "A framework for wettability-by-design", transforming empirical observation into principle-driven understanding.

The refined Table 1 confirms that chemical modification alone cannot surpass the intrinsic hydrophobic limit of flat surfaces, which remains constrained to approximately 120°. In contrast, hierarchical micro/nano-texturing enables a fundamental transition to geometry-dominated wetting, allowing metals, oxides, and polymers alike to achieve apparent contact angles ≥ 150°. This universality explains why superhydrophobic behavior has been reported across chemically diverse systems and reinforces the conclusion that surface texture, rather than molecular composition, is the decisive control parameter in this regime.

From a design perspective, these findings translate into clear and actionable rules. Superhydrophobic surfaces suitable for self-cleaning, anti-icing, and low-adhesion applications require engineered surface texture, often supplemented—but not replaced—by low-surface-energy coatings^8–9^. Conversely, superhydrophilic behavior (θ ≤ 20°) can be achieved either through high-energy flat surfaces, such as UV-activated oxides, or through porous and wicking architectures, which promote rapid liquid spreading via capillary forces. These two routes are physically distinct but converge to similar macroscopic wetting outcomes.

Beyond immediate design implications, the curated dataset provides a reliable foundation for predictive modeling and data-driven approaches, including machine-learning-assisted wettability prediction. Because the dataset spans validated chemistries, geometries, and wetting regimes, it can support model training without reliance on synthetic or inconsistent data, reducing the need for repetitive experimental measurements.

Several limitations should be acknowledged. While the dataset comprises 110 verified entries—sufficient to identify universal thresholds and regime transitions—it does not exhaustively sample all material classes, texture morphologies, or liquid properties. Parameters such as roughness scale, re-entrant geometry, droplet volume, and environmental conditions are not uniformly reported across the literature. Expanding the dataset in future work would enable deeper statistical correlations and more refined predictive capability.

Overall, the results underscore the value of long-term, carefully curated datasets in uncovering universal physical principles in surface wetting and in transforming empirical observations into transferable engineering rules.

## Conclusion

This work presents a verified 30-year dataset of static contact angles comprising 110 solid–liquid pairs, spanning polymers, metals, oxides, self-assembled monolayers, and textured or nanostructured surfaces. By filtering unreliable or inconsistent measurements, the dataset provides a robust empirical basis for identifying fundamental wetting limits.

Analysis of the refined dataset reveals universal wettability thresholds, with θ ≤ 20° corresponding to superhydrophilic behavior and θ ≥ 150° corresponding to superhydrophobic behavior. These limits demonstrate that while surface chemistry establishes baseline wettability, it is surface geometry—particularly hierarchical micro/nano-texture—that enables transitions to extreme wetting states. No chemically homogeneous flat surface in the dataset exceeds the intrinsic hydrophobic ceiling of approximately 120°, whereas textured surfaces consistently access the superhydrophobic regime.

The resulting Wettability-by-Design framework offers a practical, experiment-independent reference for engineering functional surfaces across applications including self-cleaning, anti-icing, adhesion control, condensation management, and liquid transport. By consolidating decades of verified measurements into a unified framework, this study provides a reliable platform for predictive modeling, data-driven surface engineering, and rational materials design, reducing reliance on trial-and-error experimentation.

## Data Availability

All data used or analysed during this study are included in this published article. Additional data are available from the corresponding author upon reasonable request.
